# Inhibition of TLR4 Induces M2 Microglial Polarization and Provides Neuroprotection via the NLRP3 Inflammasome in Alzheimer’s Disease

**DOI:** 10.3389/fnins.2020.00444

**Published:** 2020-05-20

**Authors:** Weigang Cui, Chunli Sun, Yuqi Ma, Songtao Wang, Xianwei Wang, Yinghua Zhang

**Affiliations:** ^1^Department of Human Anatomy, Xinxiang Medical University, Xinxiang, China; ^2^Xinxiang Key Laboratory of Molecular Neurology, Xinxiang Medical University, Xinxiang, China; ^3^Henan Key Laboratory of Medical Tissue Regeneration, Xinxiang Medical University, Xinxiang, China

**Keywords:** Alzheimer’s disease, inflammation, toll-like receptor 4, microglia, polarization

## Abstract

Accumulating evidence has indicated that activation of microglia and neuroinflammation reaction play a prominent role in Alzheimer’s disease (AD). Inhibition of toll-like receptor 4 (TLR4) has been shown to be associated with immune responses and brain damage, but its effects on AD remain unclear. This study mainly aimed to investigate the protective effect of TAK-242 (TLR4-specific inhibitor) on microglial polarization and neuroprotection in an AD mouse model and the underlying mechanisms. We found that APP/PS1 transgenic AD mice exhibited a dramatic increase in TLR4 levels concomitant with a significantly higher expression of inflammatory microglia compared to C57BL/6 wild-type mice. Furthermore, inhibition of TLR4 by TAK-242 administration significantly improved neurological function, decreased the level of Bax, and caused a significant reduction in the levels of M1-markers (iNOS and TNFα), while the expressions of M2-phenotype markers (Trem-2 and Arg-1) were increased both *in vivo* and *in vitro*. Furthermore, TAK-242 treatment enhanced BV2 microglial phagocytosis. Moreover, Aβ_25__–__35_ caused the upregulation of inflammatory cytokine production, MyD88, NF-kappaB-p65, and NLRP3, which could be ameliorated by NLRP3-siRNA or TAK-242. These findings indicated that TLR4 inhibition provided neuroprotection and promoted a microglial switch from the inflammatory M1 phenotype to the protective M2 phenotype in AD. The mechanism involved may be related to modulation of the MyD88/NF-kappaB/NLRP3 signaling pathway.

## Introduction

Alzheimer’s disease (AD) is characterized by amyloid-β (Aβ) deposition, microglial inflammation response, and neuronal loss in the diseased brain (Oliveira et al., 2018; Fahnestock and Shekari, 2019). Emerging evidence shows that microglia are usually classified into “classically” or “alternatively” activated types. Classically activated microglia (called M1 microglia) express polarization markers like inducible nitric oxide synthase (iNOS) and tumor necrosis factor alpha (TNFα), which can exacerbate neuronal damage and lead to tissue injury, whereas alternatively activated microglia (called M2 microglia) are related to the expression of polarization markers like TREM-2 and IL-10, which can promote angiogenesis and prevent neuronal death (Businaro et al., 2018). Therefore, the shift of microglia from the M1 to the M2 phenotype is of great interest.

Toll-like receptors (TLRs) are highly expressed in immune cells, belong to the pattern recognition receptors, and constitute an important part of the innate immune system. Toll-like receptor 4 (TLR4), the most studied member of the TLR family, responds to the inflammatory response and mediates inflammatory signal transduction, such as via myeloid differentiation factor88 (MyD88) and nuclear factor κB (NF-κB), through recognition of exogenous and endogenous ligands (Tao et al., 2018). TLR4 expression has been found in microglia in recent studies (Zhao et al., 2016). Previous studies have demonstrated that an increase in the expression profiles of TLR4 in retinal ganglion cells (RGCs) was induced by high glucose, and inhibition of TLR4 alleviated the inflammation and apoptosis of RGCs in high glucose (Hu et al., 2017). In addition, over-expression of TLR4 exacerbated astroglial response to LPS and increased the expression of proinflammatory cytokines such as IL-1β and TNFα (Rosciszewski et al., 2018). Several studies also found that the expression of the TLR4 and pro-inflammatory cytokines increased in AD mice and in AD patients (Huang et al., 2017; Miron et al., 2018). These observations indicate that TLR4 plays a crucial role in regulating the inflammatory response and that TLR4 inhibition may protect against inflammatory damage in the pathogenesis of AD, indicating that inhibition of TLR4 could be a novel target for AD treatment.

The cyclohexene derivative TAK-242 (Ethyl (6R)-6-[*N*-(2-chloro-4-fluorophenyl) sulfamoyl] cyclohex-1-ene-1-carboxylate), which is a specific small-molecule inhibitor of TLR4, has lower molecular weight and liposoluble capacity, which allows TAK-242 to cross the blood brain barrier (Hua et al., 2015; Tramullas et al., 2016). Investigations have shown that TAK-242 binds selectively to Cys747 in the intracellular domain of TLR4, subsequently reducing the activity of TLR4 (Matsunaga et al., 2011). The beneficial effects of TAK-242 on inflammatory reaction in diet-induced obesity were found to be associated with reduction in microglial activation and thus with the attenuation of the adverse neural effects (Moser et al., 2018). Additional studies demonstrated that inhibition of TLR4 by TAK-242 alleviated hyperalgesia and the production of IL-1β induced by acute dural inflammation in experimental migraine (Su M. et al., 2018). So far there is no information about the impact of TLR4 inhibition treatment on neuroprotection and microglial polarization state in AD. Therefore, the present study was designed to explore the effects of TAK-242 on inflammatory factor levels and microglial phenotype in AD. We further examined the role of the MyD88/NF-κB signaling pathway.

## Materials and Methods

### Animal Procedures

Adult male APP/PS1 transgenic mice and wild-type (WT) littermates, 6 months, weighing 20–25 g, were obtained from the Neurobiology and Genetics Laboratory of the Rockefeller University in New York and reproduced in Xinxiang Medical University as previously described (Cui et al., 2017). The APP/PS1 transgenic mice express a chimeric mouse/human APP650 cDNA containing the Swedish (K670M/N671L) mutations and a mutant human PS1 carrying the exon 9-deleted variant under the control of mouse prion promoter elements with a C57BL/6 background. Both transgenes are inserted at a single locus and are inherited together. APP/PS1 mice and their WT littermates used in the experiment were genotyped by polymerase chain reaction analysis of tail biopsies. All of the procedures were performed according to the NIH guidelines for the care of laboratory animals. The animal study was reviewed and approved by the Ethics Committee of Xinxiang Medical University. The mice were given free access to food and water and maintained in a temperature-regulated room at 23–25°C under an artificial 12 h light/12 h dark cycle.

### Drug Treatment

Either TAK-242 purchased from Sigma (St. Louis, MO, United States) and resuspended in 1% dimethyl sulfoxide and double-distilled water or vehicle was administered by intraperitoneal injection at a dose of 2 mg/kg/day for 28 successive days. The dose was chosen on the basis of previous *in vivo* studies reporting its anti-inflammatory profile in aldosterone-induced cardiac and renal injury (Zhang et al., 2015) and in microembolization-induced myocardial injury (Su Q. et al., 2018).

### Morris Water Maze Test

The spatial memory acquisition tests were tested by Morris water maze. In brief, a pool 100 cm in diameter and 50 cm in height was filled with water (22°C). The pool area is divided into four equal quadrants with a platform (10 cm in diameter) submerged 1 cm below the water surface. Every mouse was given four trials per day for five consecutive days. Each trial began by placing one mouse into a randomly selected quadrant, and the mouse was allowed to swim freely for a maximum of 60 s. If the mouse found the platform within 60 s, then the swimming time of the mouse will be the escape latency. If the mouse failed to locate the platform in 60 s, it was guided to the platform and allowed to stayed there for 10 s. A probe trial was conducted in which the submerged platform was removed from the pool and the mice were given 60 s to search in the pool. The swimming speed, escape latency, time spent in the target quadrant and number of target crossings were measured by the analysis-management system.

### Tissue Preparation and Immunohistochemistry

After the last injection, the brain tissues were harvested, and coronal frozen sections (20 μm thick) were cut by a freezing microtome (Leica, Nussloch, Germany). For double immunofluorescence staining, sections were blocked by 5% normal donkey serum for 1 h at room temperature and incubated with the primary antibodies; the following primary antibodies were employed: rabbit anti-TLR4 (1:200, Santa Cruz Biotechnology, Santa Cruz, CA, United States), mouse anti-Aβ (1:1,000 dilution, Sigma), mouse anti-GFAP (1:500, Sigma), mouse anti-CD11b (1:200; eBioscience, San Diego, CA, United States), and mouse anti-NeuN (1:500, Millipore, Billerica, MA, United States) for 24 h, After rinsing with PBS, sections were incubated with a mixture of Alexa Fluor^®^ 488-labeled donkey anti-rabbit IgG antibody and Alexa Fluor^®^ 594-labeled donkey anti-mouse IgG antibody (Invitrogen, Eugene, OR, United States) for 1 h at room temperature with secondary antibodies. Finally, fluorescence images of immunoreactivity were acquired using a two-photon confocal microscope (Zeiss LSM510). The area of CD11b positive reactivity was counted as we described previously (Cui et al., 2012). Briefly, five sections with a distance of 100 μm between them were counted in the whole slice under a fluorescent microscope (Olympus IX71). A threshold optical density level was obtained to discriminate staining from background by Image-Pro Plus software. The sum area of the positive reaction on a section was measured. We used morphological criteria to distinguish resting microglia from activated microglia (McClain et al., 2011). Microglia scored as resting presented long, highly ramified processes with comparatively small cell bodies, while activated microglia had shortened, swollen processes with large cell bodies. The percentage of amoeboid microglial cells over the total CD11b-positive microglial cells was calculated.

### Cell Culture and *in vitro* Experiments

BV2 microglial cell line and SH-SY5Y neuronal cells were obtained from Shanghai Institute of Cell Bank (Shanghai, China) and cultured in Dulbecco’s modified Eagle’s medium containing 10% fetal bovine serum (FBS, Gibco, United States) in a humidified atmosphere with 5% CO2. BV2 cell line (Cat# ATL03001) was not listed as a commonly misidentified cell line by the International Cell Line Authentication Committee. In our study, the BV2 cell line was authenticated by immunostaining with the microglial marker CD11b. When the BV2 cell line reached about 80% confluency, the cells were subcultured every 3 days using 0.25% trypsin to detach the cells and seeded onto six-well or 24-well plates for further experiments. Aβ25-35 peptides were purchased from American Peptide Co. (Sunnyvale, CA, United States). Before use, Aβ25-35 peptides (1 mM in sterile distilled water) were aggregated at 37°C for 3 days. TAK-242 or DMSO was added to medium for 8 h and then stimulated in the presence or absence of Aβ25-35 for 24 h.

### Primary Neuron Cultures

Primary neuronal cells were prepared from the hippocampus (HC) of newborn C57BL/6 mice (P0-P1) as previously described in our research (Cui et al., 2013). Briefly, HC fragments were isolated and brain meninges were removed. Cells were dissociated into single cells in cold 2.5 mg/mL trypsin solution and resuspended in DMEM/F12 supplemented with 10% fetal bovine serum (FBS), 0.5 mM glutamine, and 1% penicillin/streptomycin after centrifugation for 5 min. Cells were seeded onto poly-D-lysine-coated coverslips at a density of 2 × 10^6^/ml (2 ml/well). The cells were maintained in Neurobasal medium supplemented with 2% B27 and 0.5 mM glutamine in a CO_2_ incubator at 37°C for 7 days for the assay.

### Cell Viability Assays

BV2 cell line was seeded in 96-well plate at a density of 1 × 10^4^ cell per well and incubated for 24 h prior to experimental treatments. The cells were then treated with different concentrations of TAK-242 (0, 5, 10, 20, 50, 100, 200, 400, and 800 nM). Cell viability was detected by the 3-(4, 5-dimethylthiazol-2-yl)-2, 5-diphenyl- tetrazolium bromide (MTT) method as we described previously (Cui et al., 2013).

### ELISA Assay

Twelve days after TAK-242 treatment, brains from different groups of mice were collected and homogenized for ELISA analysis. BV2 cells were plated at a density of 1 × 10^5^ cells per well in a 24-well plate. Cells were then pretreated with TAK-242 (100 nM) for 8 h and stimulated with Aβ for 24 h.

The levels of iNOS, TNFα, and Arg-1 in culture medium were measured by specific ELISA kits (R&D Systems) and TREM-2 ELISA kits (RayBiotech, Norcross, GA, United States) according to the manufacturer’s instructions.

### Western Blot Assays

After the last injection, the prefrontal cortex (PFC) and hippocampus (HC) brain tissues were harvested. For the BV2 cell line, BV2 cells were seeded onto a six-well plate at a concentration of 2 × 10^6^ cells per well, and the cells were pretreated with TAK-242 (100 nM) or DMSO for 8 h. They were then stimulated with Aβ (25 μM) for 24 h, and then the BV2 cells were collected. Proteins from the PFC and HC tissues and cells were lysed and extracted using RIPA lysis buffer (20 mM Tris-HCl, pH 7.4, 150 mM NaCl, 5 mM EDTA, 1% Non-idet P40) containing protease inhibitor (Sigma, St. Louis, MO, United States); protein concentration was measured using a BCA protein assay kit. Subsequently, 10 μg of protein in 5 μl of sample buffer was added to a 10% polyacrylamide gel. Protein was separated by SDS/PAGE and was transferred onto a polyvinylidene difluoride membrane then incubated with 5% non-fat milk for 1 h at room temperature. The membrane was incubated with the following primary antibodies overnight at 4°C: rabbit anti-MyD88 (1:1,000, Santa Cruz, CA, United States), rabbit anti-NF-κB p65 (1:1,000, Santa Cruz, CA, United States), rabbit anti-NLRP3 (1:1,000; Abcam, United Kingdom), rabbit anti-Bax, 1:5,000 (Sigma-Aldrich, St. Louis, MO, United States), and rabbit anti-GAPDH, 1:5,000 (Sigma-Aldrich, St. Louis, MO, United States). After washing three times with TBST (10 mMTris-Cl, 150 mM NaCl, and 0.1% Tween 20), membranes were incubated with 5% skimmed milk containing 0.1% Tween and horseradish peroxidase-conjugated secondary antibody for 2 h at room temperature (Pierce, Rockford, IL, United States). Immunostained bands were detected by ECL, and the integrated intensity of protein bands was normalized by using GAPDH by densitometry with gel documentation system software (Bio-Rad).

### Conditioned Medium Collection, SH-SY5Y Cell Induction, and Primary Neuron Induction

Conditioned medium from BV2 cells was collected at desired time points after each group treatment and then filtered through 0.45-μm filters and quickly frozen at −80°C for further SH-SY5Y cell and primary neuronal cell treatments. Briefly, BV2 cells were plated on poly-D-lysine-coated slides and were pretreated with TAK-242 (100 nM) or DMSO for 8 h. They were then stimulated with Aβ (25 μM) for 24 h, and then the medium was collected as conditioned medium. SH-SY5Y cells and primary neuronal cells were exposed to conditioned medium from BV2 microglial cells for 8 h. The level of Bax and DNA fragmentation in SH-SY5Y was then measured.

### Quantification of DNA Fragmentation

DNA fragmentation was detected by a Cell Death Detection ELISAPLUS kit (Roche Diagnostics) according to the manufacturer’s protocol. Briefly, the cells or tissues were collected and washed with D-Hanks solution after each drug treatment, incubated with lysis buffer (200 μl) for 30 min at 37°C, and then centrifuged at 200 × *g* for 10 min at 4°C. The supernatant from each well (20 μl) was incubated in a streptavidin-coated microplate with a mixture of anti-histone-biotin and anti-DNA-peroxidase. The apoptotic nucleosomes were captured by the anti-histone-biotin antibody via histone component and the anti-DNA-peroxidase, which were bound to the DNA part of the nucleosomes. After removing the free antibodies, the amount of peroxidase was quantified by 2,2’-azinobis (3-ethylbenzthiazoline-6-sulfonic acid) (ABTS). The absorbance at 405 nm was measured using a microplate reader.

### Immunocytochemistry and Phagocytosis Assay

For immunofluorescence staining, the BV2 cells were seeded into a 12-well plate (2 × 10^5^ cells/well). When they had grown to 80% confluence, the BV2 cells were rinsed with PBS, fixed with 4% paraformaldehyde (PFA) solution for 30 min at room temperature, and then washed thoroughly with PBS. They were then incubated with mouse anti-CD11b (1:200; eBioscience, San Diego, CA, United States) at 4°C overnight and then incubated in Alexa Fluor^®^ 594-labeled donkey anti-mouse IgG antibody (Invitrogen, Eugene, OR) for 2 h. Subsequently, after washing, nuclei were stained with DAPI for 5 min. The phagocytic ability of BV2 microglial cells was measured by the uptake of fluorescent microspheres (latex beads, carboxylate-modified polystyrene, fluorescent yellow aqueous suspension, Sigma). The pure microglia were seeded into a 12-well plate (1 × 10^6^ cells/well) and were stimulated with Aβ for 24 h with or without TAK-242 pretreatment for 8 h. For phagocytosis measurement, 4 μL/mL of carboxylate-modified polystyrene latex beads was added to each well and maintained for 2 h at 37°C. The cells were washed three times with D-Hanks to remove the free latex beads in the medium and then fixed with 4% PFA. The cells were incubated with the mouse anti-CD11b antibodies and Alexa Fluor^®^ 594-labeled donkey anti-mouse IgG second antibodies, and nuclei were stained with DAPI for 5 min. Three random fields per well were preserved, and microscopic images of immunoreactivity were captured using a computer-controlled fluorescent microscope (Olympus IX71) or two-photon confocal microscope (Zeiss LSM 510).

### RNA Interference and Transfection

BV2 cells either with NLRP3 siRNA (Ribo were transfected Biotechnology, Shanghai, China, sense, 5′-GCUUCAGCCACAUGACUUUTT-3′, antisense 5′-AAAGUCAUGUGGCUGAAGCTT-3′) or control siRNA (sense, 5′-UUCUCC GAA CGU GUC ACG UTT-3′, antisense, 5′-ACG UGA CAC GUU CGG AGA ATT-3′) by using Lipofectamine LTX transfection reagent following the manufacturer’s protocol. Cells were analyzed after 24-h transfection in indicated assays.

### Statistical Analysis

Results were expressed as the mean ± standard deviation (SD) and analyzed by using SPSS 19.0 software. Comparisons between two groups were analyzed by Student’s *t*-test. When there were three groups or more, data were subjected to one-way ANOVA with Bonferroni correction. For the Morris water maze test, two-way repeated-measures ANOVA followed by a *post hoc* Bonferroni’s test was used. *P* < 0.05 was set as indicating statistical significance.

## Results

### Alteration of TLR4 in APP/PS1 Transgenic Mice

**FIGURE 1:**
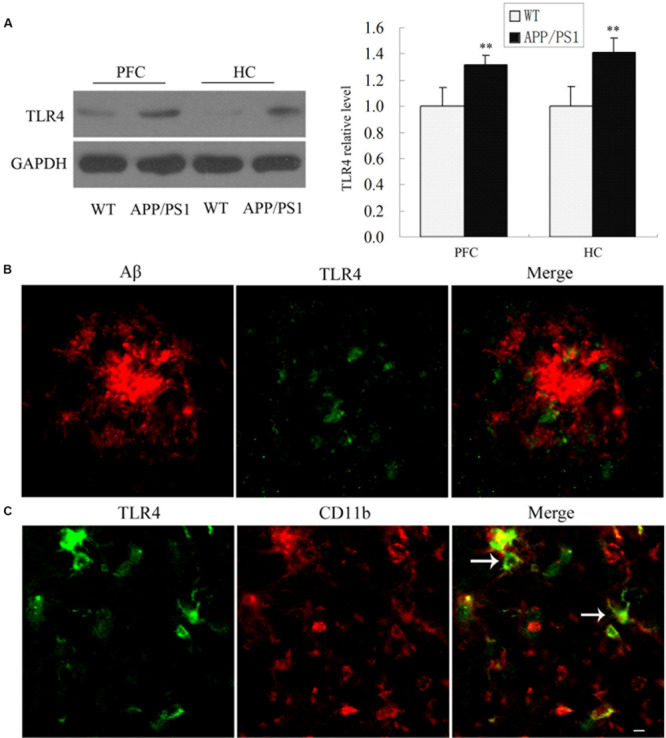
TLR4 expressions were increased in the APP/PS1 Transgenic Mice. TLR4 levels in 6-month-old APP/PS1 transgenic and WT mice were shown by Western blot **(A)**. Confocal analysis for Aβ (red) and TLR4 (green) revealed that Aβ deposition was surrounded by TLR4-positive cells in APP/PS1 transgenic AD mice **(B)**. TLR4 (green) colocalized with CD11b (red) **(C)**, which is indicated by an arrow. Scale bar: 20 μm. GAPDH was used as a loading control, and the densitometry was quantified. Data were analyzed with Student’s *t*-test. Data are expressed as mean ± SD, *n* = 5 for each group (^∗∗^*P* < 0.01 compared with the WT mice).

To determine whether TLR4 was involved in the inflammatory reaction, we first measured TLR4 protein expression in APP/PS1 transgenic mouse at 6 months of age compared with WT mice. As shown in Figure 1A, APP/PS1 transgenic mice showed an increased TLR4 expression compared to the WT group. Most interestingly, TLR4-positive cells were observed predominantly at the sites of Aβ deposition in APP/PS1 transgenic AD mice (Figure 1B). Moreover, we also observed that TLR4 was expressed in CD11b-positive cells by double-immunostaining in the PFC of APP/PS1 AD mice (Figure 1C).

### Modulation of Microglial Polarization in APP/PS1 Transgenic Mice After TAK-242 Treatment

**FIGURE 2:**
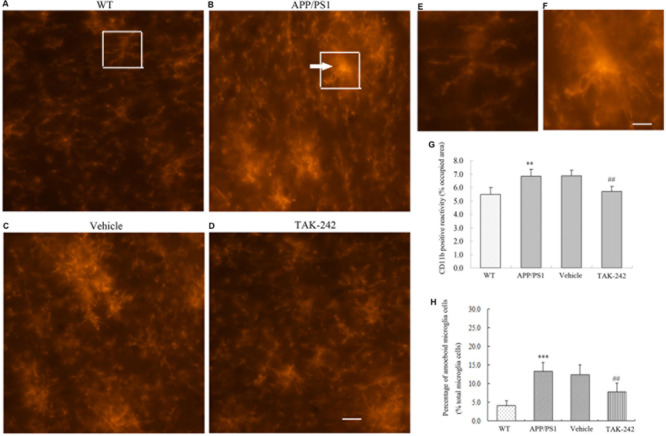
Microglial morphological changes in the PFC in APP/PS1 AD mice and its regulation by TAK-242 treatment. Representative immunofluorescence staining images show CD11b-positive microglia in the PFC of WT mice **(A)** and APP/PS1 transgenic mice **(B)**. Morphological changes were suppressed by treatment with TAK-242, as shown in the immunofluorescence staining of vehicle **(C)** and TAK-242 **(D)** treatments. Hypertrophic morphology with thick microglial processes is indicated by an arrow. Panels **(E)** and **(F)** are magnifications of the rectangular region indicated in parts **A** and **B**, Scale bar: 5 μm. The area of CD11b positive reactivity is presented **(G)**. The percentage of amoeboid microglial cells over the total microglial cells for each experimental group is presented **(H)**. Data were analyzed with one-way ANOVA followed by *post hoc* Bonferroni test analysis. Data are expressed as mean ± SD, *n* = 5 for each group (^∗∗^*P* < 0.01, ^∗∗∗^*P* < 0.001 compared with the WT mice group. ^##^*P* < 0.01 compared with the Vehicle-treated control group).

Microglia are the resident immune cells and the first responders to any type of injury in brain. We discovered that TLR4 was expressed in CD11b-positive microglial cells in AD mice. We therefore examined the microglial cell morphology and density after TAK-242 treatment. Compared with the WT mice, the average area of cells of CD11b-positive microglia was significantly increased in APP/PS1 AD mice (Figure 2G). Visual observation found that the microglial cells transformed from the ramified shape with long, thin complex processes in WT mice to the amoeboid shape with short, thick processes and a large soma in APP/PS1 transgenic AD mice (Figures 2A–F). Furthermore, TAK-242 treatment significantly attenuated the microglial activation as determined by the decreased CD11b expression and the average area of cells (Figure 2G). We also found that CD11b-positive microglia transformed from the amoeboid activated state (Figure 2C) to the more ramified resting state in terms of morphology after TAK-242 treatment (Figure 2D). The morphological changes of microglial cells were also examined. As shown in Figure 2H, the APP/PS1 mice displayed a higher percentage of amoeboid microglial cells than the WT mice, indicating the activation of microglial cells in the APP/PS1 mice. Nevertheless, when APP/PS1 mice were treated with TAK-242, the morphological changes were inhibited, evidenced by the decreased percentage of amoeboid microglial cells compared with the vehicle-treated group.

**FIGURE 3:**
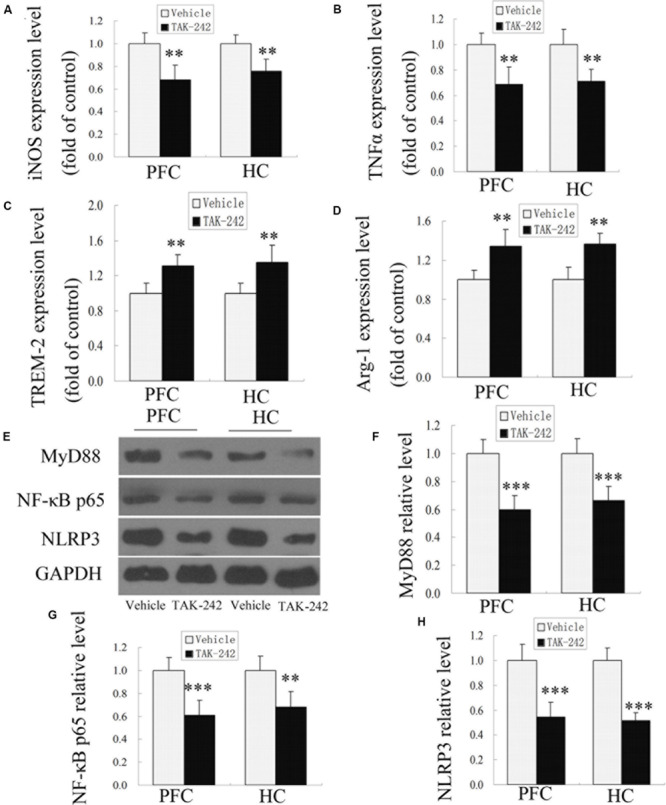
M1- and M2-microglia associated markers and MyD88, NF-κB p65, and NLRP3 signaling were altered after TAK-242 treatment in APP/PS1 transgenic mice. ELISA analysis for the M1-microglia-associated markers iNOS and TNFα **(A,B)** and the M2-microglia-associated markers TREM-2 and Arg-1 **(C,D)**. TAK-242 decreased the expression of MyD88, NF-κB p65, and NLRP3 **(E–H)**. GAPDH was used as a loading control. Data were analyzed with Student’s *t*-test. Data are expressed as mean ± SD, *n* = 5 for each group (^∗^significant as compared with vehicle-treated control group, ^∗∗^*P* < 0.01, ^∗∗∗^*P* < 0.001).

Microglia play dual roles in neuroinflammation, depending on the microglial phenotype. To confirm whether TAK-242 administration could induce microglial polarization switching from the classical pro-inflammatory M1 phenotype to the alternatively activated M2 phenotype, ELISA was used to detect the expression levels of M1 phenotype makers (iNOS and TNFα) and M2 phenotype markers (TREM-2 and Arg-1) in APP/PS1 transgenic mice. The results showed that treatment with TAK-242 caused a significant reduction in the levels of M1-markers (iNOS and TNFα) (Figures 3A,B), while the expression of M2-phenotype markers (TREM-2 and Arg-1) was increased in comparison to the vehicle-treated group (Figures 3C,D), which indicated that inhibition of TLR4 promoted M1–M2 switch.

### Effects of TLR4 Inhibition on MyD88/NF-κB and NLRP3 Signaling-Related Protein in APP/PS1 AD Mice

In order to investigate the possible mechanism of TLR4 inhibition in inflammatory response, we examined the expression of MyD88, NF-κB p65, and NLRP3 proteins by Western blot analysis. As shown in Figures 3E–H, compared with the vehicle-treated group, TAK-242 treatment induced downregulations of MyD88, NF-κB p65, and NLRP3 in APP/PS1 AD mice. These results suggested that the alleviation of inflammation response by TAK-242 was associated with reduced levels of MyD88/NF-κB and NLRP3 inflammasome signaling.

### TLR4 Inhibition Ameliorated Learning and Memory Impairment, Lowered Aβ Deposition, and Inhibited Neuronal Apoptosis in APP/PS1 Transgenic AD Mice

**FIGURE 4:**
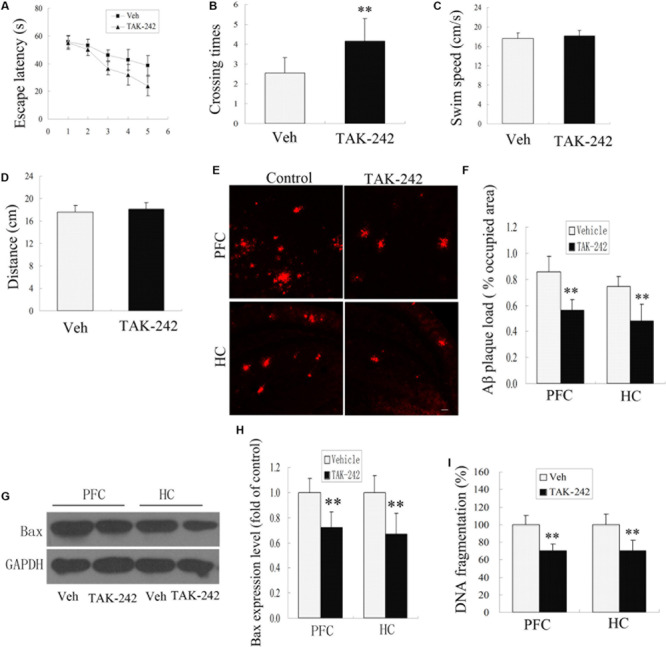
TLR4 inhibition treatment ameliorated impaired performance, decreased Aβ deposition, and exerted an anti-apoptosis effect in AD mice. The vehicle-treated group exhibited longer escape latencies to reach the hidden platform compared with TAK-242-treated mice **(A)**, *P* < 0.05 by repeated-measure two-way ANOVA (Veh, *n* = 13; TAK-242, *n* = 13). The number of times of crossing the platform are shown **(B)**, ^∗∗^*P* < 0.01 compared with the vehicle-treated group (Student’s *t*-test). No differences in the swim speeds or total distance **(C,D)** were found in TLR4 inhibition group compared with vehicle-treatment group. TAK-242 reduced the amyloid load in PFC and HC as the percentage of surface coverage **(E,F)**, Scale bar 60 μm. TLR4 inhibition decreased the expression of Bax and the extent of DNA fragmentation **(G–I)**, Data were analyzed with Student’s *t*-test. Data are expressed as mean ± SD. ^∗∗^*P* < 0.01, compared with vehicle-treated control.

The APP/PS1 transgenic AD mice are well known to develop Aβ-associated cognitive deterioration at the age of 6 months (Jankowsky et al., 2004). To examine whether TAK-242 affected cognitive ability, learning and memory capacity were assessed by the Morris water maze test. As shown in Figures 4A,B, the Morris water maze results revealed that TAK-242 treated mice had a significant decrease in latency in finding the hidden platform, and the number of crossings in the TAK-242-treated group was significantly higher than in the vehicle-treated group (Figures 4A,B). It should be noted that there was no significant difference in the mean speed and total distance between any of the groups (Figures 4C,D), suggesting that the differences in escape latencies were not due to differences in locomotor ability among different groups. The results indicated that TAK-242 treatment improved the learning and memory functions in AD mice.

Cognitive impairment is correlated with Aβ deposits in the PFC and HC in APP/PS1 AD mice (Jankowsky et al., 2004). In this model, plaques are known to be well established and visible at 6 months of age. We next tested whether TLR4 inhibition by TAK-242 affected the deposition of Aβ. Immunohistochemical staining for Aβ showed a striking difference in the percentage of the area occupied by plaque load in the PFC and HC between the TAK-242-treated group and the vehicle-treated group (Figures 4E,F).

More and more evidence is showing that apoptosis is involved in the neuronal loss in AD. In order to investigate the effect of TLR4 inhibition on the anti-apoptosis effects, we examined the expression levels of apoptosis-related protein Bax and the extent of DNA fragmentation. Western blot assay showed that Bax protein levels decreased significantly in the TAK-242 treatment group compared with the vehicle-treatment control group (Figures 4G,H). The extent of DNA fragmentation also decreased after treatment with TLR4 inhibitor TAK-242 (Figure 4I).

### Effects of TAK-242 Treatment on Inflammation Expression, Phagocytic Efficiency, and M1/M2 Polarization in Response to Aβ Treatment in BV2 Microglial Cells

**FIGURE 5:**
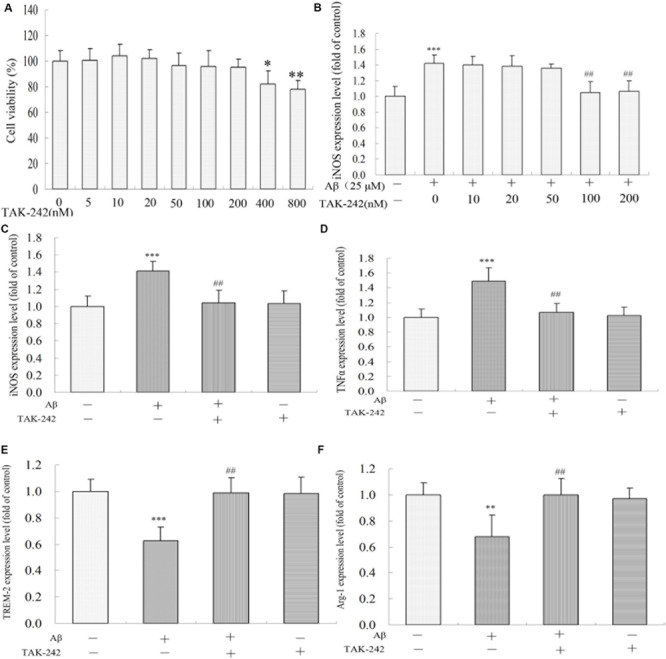
TAK-242 regulated Aβ-induced M1 and M2 inflammatory microglia polarization. BV2 microglia were treated with various concentrations of TAK-242 for 24 h, and the cell viability was then measured by MTT assays **(A)**. The optimal dose of TAK-242 in inhibiting Aβ-induced inflammation response was analyzed by ELISA assay **(B)**. The expressions of iNOS, TNFα, TREM-2, and Arg-1 **(C–F)** were measured by ELISA kit. Data were analyzed with Student’s *t*-test or one-way ANOVA followed by *post hoc* Bonferroni test analysis. All the experiments were repeated three times (*n* = 5). Data are expressed as mean ± SD (^∗^*P* < 0.05, ^∗∗^*P* < 0.01, ^∗∗∗^*P* < 0.001 compared with the Vehicle-treated control group. ^##^*P* < 0.01 compared with the Aβ25–35 (25 μM)-treated group).

To determine the most effective concentration of TAK-242 in BV2 microglial cells, the cytotoxicity of TAK-242 on BV2 microglia was determined by MTT assay. BV2 microglia were incubated with various concentrations (0, 5, 10, 20, 50, 100, 200, 400, and 800 nM) of TAK-242 for 8 h. As shown in Figure 5A, the viability of BV2 microglial cells was not affected by TAK-242 at concentrations below 200 nM, while at concentrations of 400 and 800 nM, TAK-242 significantly decreased cell viability. To further investigate the effective dose of TAK-242 in inhibiting Aβ-induced inflammation reaction, BV2 microglial cells were treated with 25 μM Aβ for an additional 24 h followed the application of TAK-242 for 8 h. The results showed that 100 nM TAK-242 dramatically inhibited the expression of iNOS (Figure 5B). Therefore, TAK-242 at a concentration of 100 nM was used in the subsequent studies.

We also investigated the role of TLR4 inhibition in Aβ-induced inflammation reaction and microglial phenotype in BV2 microglia. As shown in Figures 5C,D, Aβ increased the levels of M1 phenotype markers (iNOS and TNFα) but decreased the M2 phenotype markers (TREM-2 and Arg-1) (Figures 5E,F). However, these M1 phenotype proinflammatory mediators were significantly inhibited by TAK-242 treatment. Further, TAK-242 pretreatment markedly increased the effect of Aβ on M2 phenotype markers in BV2 cells.

**FIGURE 6:**
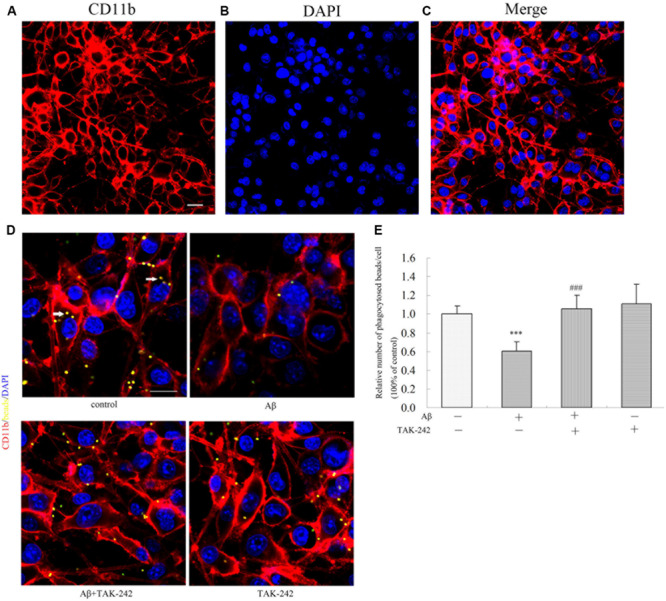
TAK-242 promoted microglial phagocytosis. The purity of the BV2 cell line was identified by immunostaining with an CD11b antibody specific to microglia **(A–C)**. Scale bar: 20 μm. Compared with Aβ-treated control cells, the effect of TAK-242 on microglial phagocytosis was assessed by the number of fluorescent microbeads (yellow) in cells. CD11b-labeled microglia are shown in red, and DAPI-labeled cell nuclei are shown in blue. Fluorescent microbeads in yellow are indicated by an arrow **(D,E)**. Scale bar: 20 μm. Data were analyzed with one-way ANOVA followed by *post hoc* Bonferroni test analysis. All the experiments were repeated three times (*n* = 5). Data are expressed as mean ± SD (^∗∗∗^*P* < 0.001 compared with the Vehicle treated group. ^###^*P* < 0.001 compared with the Aβ25–35 (25 μM)-treated group).

The purity of the BV2 cell line was authenticated by immunostaining with a CD11b antibody specific to microglia. We found that approximately 97% of total culture cells were identified as CD11b-positive microglial cells (Figures 6A–C). Phagocytosis is one of the most important functions of BV2 in the inflammation immune response. To assess whether TAK-242 has an influence on the phagocytic activity of BV2 microglial cells, BV2 microglial cells were stimulated with Aβ in the presence or absence of TAK-242. Then, the BV2 cells were incubated in the presence of carboxylate-modified polystyrene fluorescent yellow latex beads. We found that the phagocytic function in the Aβ + TAK-242 exposed group was much higher than that in the Aβ-treated group (Figures 6D,E).

**FIGURE 7:**
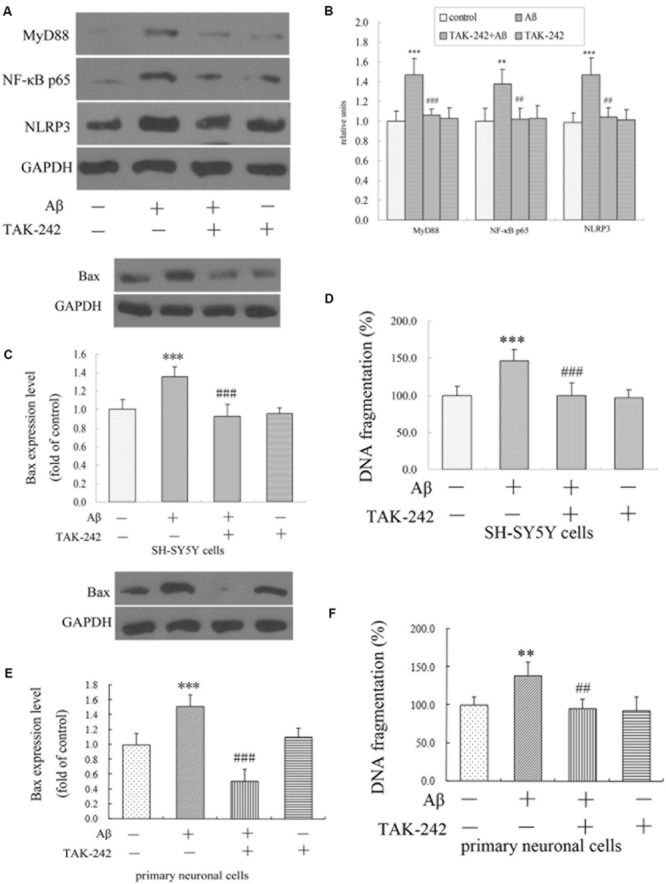
TAK-242 suppressed Aβ-induced MyD88/NF-κB p65 and NLRP3 inflammasome activation and Aβ-induced apoptosis in BV2 microglial cells. TAK-242 suppressed the expression of MyD88 NF-κB p65 and NLRP3 by Western blot **(A,B)**. TAK-242 treatment decreased the level of Bax and DNA fragmentation in SH-SY5Y cells **(C,D)** and in primary neuronal cells **(E,F)**. GAPDH was used as a loading control. Data were analyzed with one-way ANOVA followed by *post hoc* Bonferroni test analysis. All the experiments were repeated three times (*n* = 5). Data are expressed as mean ± SD (^∗∗^*P* < 0.01, ^∗∗∗^*P* < 0.001 compared with the Vehicle treated group. ^##^*P* < 0.01, ^###^*P* < 0.001 compared with the Aβ25–35 (25 μM)-treated group).

The results *in vivo* and *in vitro* indicated that TAK-242 had great inhibitory effects on microglia inflammation reaction and microglial phenotype. We next explored the possible mechanism involved. Compared with the vehicle-control group, the Aβ-treated group exhibited a significant increase in the levels of MyD88, NF-κB p65, and NLRP3 (Figure 7). Compared with the Aβ-treated group, the protein levels of MyD88, NF-κB p65, and NLRP3 in the Aβ + TAK-242 group were decreased significantly (Figure 7). The above results suggested that the MyD88/NF-κB signaling pathway participated in the inhibition of TLR4 in cytokine secretion. More importantly, the enhanced level of NLRP3 was reversed by administration of TAK-242 (Figures 7A,B), suggesting that TLR4 might serve as the upstream signal of NLRP3 in Aβ-induced inflammation reaction.

### The Effect of TLR4 Inhibition on Microglia-Mediated Neurotoxicity

We have shown that the M1/M2 polarization of microglia was triggered by TLR4 inhibition. However, we did not know how neuronal apoptosis was affected by microglia polarization. In this section, we cultured SH-SY5Y neuronal cells and the primary neuronal cells with culture medium of BV2 cells treated by TAK-242 and/or Aβ25–35, and the levels of apoptosis-related protein Bax and of DNA fragmentation of SH-SY5Y cells were investigated. As shown in Figures 7C–F, when the conditioned medium from Aβ-stimulated microglia was added to cultured SH-SY5Y cells and primary neuronal cells, the levels of Bax and DNA fragmentation were dramatically increased compared with the control conditioned medium group. However, the conditioned medium from BV2 cells with TAK-242 pretreatment prior to Aβ stimulation reduced the cell apoptosis, which provided evidence that TLR4 inhibition exerted a neuroprotective effect by suppressing microglial activation and subsequent neurotoxicity.

### Silence of NLRP3 Attenuated Inflammatory Factor Expression Induced by Aβ Treatment

**FIGURE 8:**
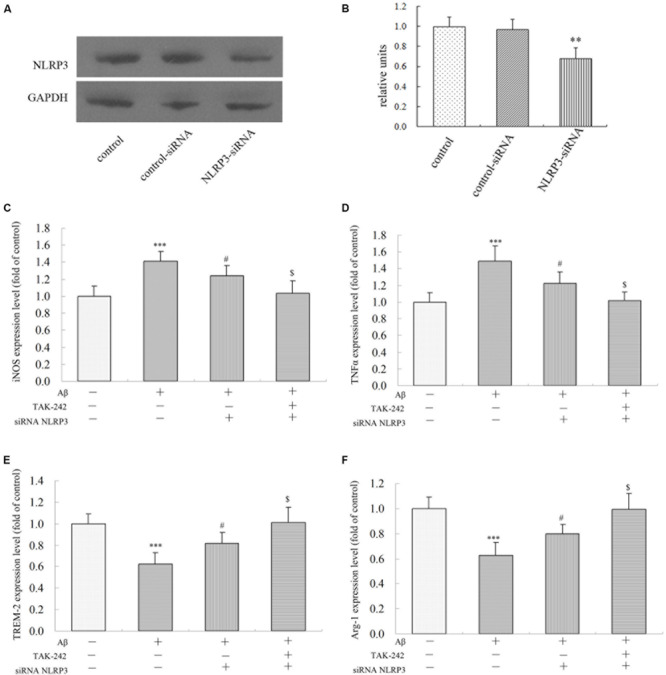
Effects of NLRP3 Gene Silencing on Aβ-Induced M1 and M2 inflammatory microglial polarization. **(A,B)** BV2 cells transfected with control or NLRP3-specific siRNA for 48 h. The relative levels of NLRP3 to GADPH protein expression were determined by Western blot. Knockdown of NLRP3 mRNA by NLRP3 siRNA in BV2 microglia significantly mitigated Aβ-induced levels on M1 markers (iNOS and TNFα) **(C,D)**, and the Aβ-induced levels of iNOS and TNFα were also mitigated by TAK-242 treatment. Consistent with these findings, increases in TREM-2 and Arg-1 expressions were facilitated by treating siRNA-NLRP3 **(E,F)** in cultured supernatant, as measured by ELISA. Data were analyzed with one-way ANOVA followed by *post hoc* Bonferroni test analysis. All the experiments were repeated three times (*n* = 5). Data are expressed as mean ± SD (^∗^significant as compared with the vehicle-treated group, ^∗∗∗^*P* < 0.001. ^#^significant as compared with the Aβ25-35 (25 μM)-treated group, ^#^*P* < 0.05. ^$^Significant as compared with the Aβ + siRNA NLRP3-treated group, ^$^*P* < 0.05).

To investigate the role of the NLRP3 inflammasome in Aβ-induced changes in inflammatory factor release, we transfected either with a non-targeting siRNA or siRNA against NLRP3 in BV2 microglial cells. As shown in Figures 8A,B, transfection with NLRP3 siRNA dramatically reduced the relative level of NLRP3 expression in BV2/siRNA-NLRP3 cells compared with that in the control-siRNA cells. Pretreatment with NLRP3-siRNA for 8 h before Aβ treatment dramatically reversed the upregulation of M1 phenotype markers (iNOS and TNFα) (Figures 8C,D). Likewise, the expression of M2 phenotype markers (TREM-2 and Arg-1) (Figures 8E,F) decreased by Aβ was concurrently increased by NLRP3-siRNA. Moreover, TAK-242-mediated inhibition of iNOS and TNFα was significantly facilitated by treating with siRNA-NLRP3 (Figures 8C,D). Consistent with the iNOS protein expression, under Aβ stimulation, the changes in M2 markers mediated by TAK-242 (Figures 8E,F) were also facilitated by siRNA-NLRP3. These results from NLRP3 gene silencing support that the effectiveness of treatment with TAK-242 may be partly due to inhibition of NLRP3 activation.

## Discussion

Microglial activation is believed to play a vital role in the development of AD (Latta-Mahieu et al., 2018). There are accumulating data showing that TLR4 is closely related to microglial activity (Zhou et al., 2019). However, the role of TLR4 in microglial phenotype polarization is not clear. We demonstrated that the upregulation of TLR4 was closely related to microglial activity and neuroinflammation. Especially, a TLR4 signaling inhibitor, TAK-242, could exert a protective effect on impairment in APP/PS1 transgenic AD mice, act as a microglia modulator, suppress MyD88/NF-κB/NLRP3 activation, and reverse microglial phenotype polarization from M1 to M2 *in vitro* and *in vivo*. Therefore, TAK-242 may be a therapeutic agent for AD. To the best of our knowledge, this study is the first to examine the activation of TLR4 in microglial polarization and its protective effect in AD.

In AD, microglial cells are activated, leading to the production of a large number of inflammatory factors and inducing neuronal injury. Therefore, inhibiting inflammatory reaction can significantly alleviate Aβ-induced injury and reduce neurological deficits (Latta-Mahieu et al., 2018). Toll-like receptors play an important role in the immune defense in various tissues including the brain (Zhou et al., 2019). In a recent study, significantly higher percentages of inflammatory monocytes/macrophages, including TLR2 and TLR4, were seen in AD (Saresella et al., 2014). Our results show that there was a marked increase in TLR4 expression in AD mice compared with WT mice. We also investigated the localization of TLR4 in AD. Our results revealed that TLR4-positive cells were localized in microglia, consistent with previous studies that showed that TLR4 was expressed in microglia (Li et al., 2014; Zhao et al., 2016). All of these results indicated that the TLR4 signaling pathway may be associated with AD and that inhibiting TLR4 may decrease the activation of microglia and thereby reverse the microglial phenotype.

TAK-242, a selective inhibitor of TLR4, binds selectively to TLR4, has a low molecular weight, can disrupt the interaction of TLR4 with adapter molecules, and can then inhibit transduction of its downstream signaling pathway. Because of its high liposolubility and a low molecular weight, TAK-242 has the capacity to pass through the blood–brain barrier (Hua et al., 2015). The specificity of TAK-242 has been confirmed by many studies. TAK-242 has been found to be safe in humans for the treatment of sepsis (Yang et al., 2018). In addition, administration of TAK-242 attenuated the degree of acute kidney injury during the ischemic reperfusion process (Mohammad et al., 2018). Shibata et al. (2017) showed that TAK-242 inhibited autoinflammatory symptoms in DITRA. Moreover, TAK-242 protected against apoptosis and improved coronary microembolization induced in rats (Wang et al., 2017). However, whether TAK-242 has anti-inflammation effects and neuroprotective effect in AD brain has remained unknown. In this study, we observed a significant decrease in the expression levels of Bax and DNA fragmentation and of the downstream proteins of TLR4, MyD88 and NF-κB, after TAK-242 treatment, in accordance with previous studies, which showed that TLR4/MyD88/NF-κB signaling participated in the inflammatory response (Xie et al., 2016; Zhou et al., 2017). In BV2 microglial cells, we indicated that Aβ significantly increased the expressions of MyD88, NF-κB p65, and NLRP3 and the levels of iNOS. However, these upregulations were reversed by co-culturation with TAK-242. These results demonstrated that the MyD88/NF-κB signaling pathway was involved in the anti-inflammation effects of TAK-242.

Many studies have suggested that M1/M2 polarization of microglia play an important role in the response to neuroinflammation (Fiala et al., 2018; Maezawa et al., 2018). Our results indicated that TAK-242 treatment resulted in an increase in CD11b-positive cells expressing M2 markers and a significant decrease in the level of M1 markers in AD transgenic model and that microglia exhibited more ameboid-shaped microglia with enlarged soma and thickened processes, highlighting the protective role of TAK-242. To learn more about the potential effects of TAK-242 on neurodegenerative diseases, we carried out experiments by Morris water maze and through immunofluorescence staining. We found that TAK-242 treatment markedly ameliorated cognitive deficit, decreased Aβ deposition, and suppressed microglial morphological changes in AD mice. Aβ has been found to be a strong inducer of M1 microglia (O’Halloran et al., 2014; McCarthy et al., 2016). Therefore, we used Aβ as a proinflammatory stimulant for *in vitro* study. In our experiments, we found that the protein expression of M1 markers was upregulated by Aβ stimulation, in line with *in vivo* results. In contrast, M2-like microglia have the ability to secrete cytokines, as demonstrated by the expression of TREM-2 and Arg-1, which are involved in the suppression of neuroinflammation (Arcuri et al., 2017; Aryanpour et al., 2017). In addition, we found that the expressions of M2 markers were significantly promoted by TAK-242 therapy. More interestingly, in our study, TAK-242 exaggerated the intake of microbeads. Taken together, we speculated that TAK-242 promoted microglial phagocytosis of Aβ, probably through the induction of M2 activation. Microglial activation plays a key role in neuronal death through neurotoxic microglial–neuronal interactions by releasing inflammatory mediators in the pathogenesis of AD (Zhou et al., 2019). In our study, the conditioned medium from Aβ-stimulated microglia was potently toxic to SH-SY5Y neuronal cells according to Western blot testing. However, TAK-242 pretreatment could decrease the level of Bax in and exert neuroprotective effects on SH-SY5Y neuronal cells exposed to conditioned medium from Aβ-stimulated microglia. All of these results demonstrated that inhibition of TLR4 may have a neuroprotective function via an anti-inflammatory mechanism mediated by the suppression of microglial activation and the release of cytotoxic factors, which could probably be further developed for the treatment of neuroinflammatory conditions in AD. To our knowledge, this is the first study to demonstrate that TAK-242 can promote microglial polarization from the inflammatory M1 state to the “anti-inflammatory, repair” M2 state. Indeed, several studies have appeared showing that a switch from M1 to M2 microglial phenotype exerts anti-inflammatory effects in several neurodegenerative diseases (Lu et al., 2018). For instance, Magnesium Lithospermate B promoted a phenotypic switch from the M1 to the M2 phenotype to suppress neuroinflammation in LPS-injected mice (Tai et al., 2018). Acute hypoxia modulated the microglia M1/M2 subgroup profile in AD (Zhang et al., 2017). Furthermore, Interferon regulated microglial M1/M2 activation after ischemic stroke in mice (Al et al., 2018). Collectively, these studies and our own findings further indicate that M2 microglia are a healthier phenotype with enhanced phagocytic capacity. Furthermore, we demonstrated that M2 microglia promote the survival of neurons under Aβ-induced inflammatory conditions. All of these results suggest that TAK-242 treatment may induce microglial switch from the inflammatory M1 phenotype to the protective M2 phenotype and increase the phagocytic function in AD. Therefore, the early switch from the inflammatory M1 phenotype to the protective M2 phenotype through inhibition of TLR4 may represent an endogenous effort to inhibit inflammation and restrict brain damage (Hu et al., 2012).

Moreover, secretion of inflammatory cytokines requires inflammasome activation. The NLRP3 inflammasome has been found to play an important role in microglial activation (Tan et al., 2013). Knockout of NLRP3 resulted in the downregulation of inflammatory cytokine production in Staphylococcus aureus infection (Ma et al., 2018). A recent study has found that knockdown of NLRP3 alleviates inflammatory cytokine in human renal tubular cells (Song et al., 2018). According to these studies, we explored the level of NLRP3 inflammasome in AD and in BV2 microglia to elucidate whether the anti-inflammatory effect of TAK-242 was associated with NLRP3 inflammasome. Our results showed that a significant upregulation of NLRP3 was found in AD mice and in BV2 microglial cells and that TAK-242 treatment significantly inhibited the level of NLRP3. Furthermore, the inhibition of iNOS and the increase of M2 marker (TREM-2) mediated by TAK-242 were also facilitated by transfecting siRNA-NLRP3. All of these results demonstrate that TAK-242 alleviates Aβ-induced inflammation response and polarization of microglia via inhibiting the NLRP3 inflammasome signaling pathway.

However, this study has some limitations that should be considered. First, in our *in vitro* studies, we chose to use the BV2 microglial cell line for the reason that BV2 was a microglia-like cell line, which have been shown to mimic primary microglia very closely and have been used as a substitute for primary microglia in *in vitro* study (Henn et al., 2009; Shi et al., 2013). Nevertheless, confirmation of results in primary microglial cells would be more reasonable and advantageous. Second, the signaling pathway through which the inhibition of TLR4 exerts anti-inflammation effects was not completely revealed. For example, the roles of JNK, MAPK, and other downstream signaling mediators in TLR4 inhibition were not examined in our study. Further research on the relationship between TLR4 and inflammation response in primary microglial cells is expected.

## Conclusion

We demonstrate that TLR4 expression is increased in AD mice. Inhibition of TLR4 shifts the microglial polarization from M1 to M2 and protects neuronal cells against the cytotoxicity of activated BV2 microglial cells via the downregulation of MyD88/NF-κB and NLRP3 signaling in AD.

## Data Availability Statement

The data sets used and/or analyzed during the current study are available from the corresponding author on reasonable request.

## Ethics Statement

The animal study was reviewed and approved by the Ethics Committee of Xinxiang Medical University.

## Author Contributions

WC and YZ conceived and designed the experiments. SW, CS, and YM performed the research and analyzed the data. WC wrote the manuscript. XW contributed to the manuscript revision. All authors read and approved the final manuscript.

## Conflict of Interest

The authors declare that the research was conducted in the absence of any commercial or financial relationships that could be construed as a potential conflict of interest.
